# Life Stage-Specific Burdens and Impacts of Gastrointestinal Nematodes in Beef Cattle in the United States: A Review of Diagnostics, Impacts on Productivity, and Immune Response

**DOI:** 10.3390/vetsci13030210

**Published:** 2026-02-24

**Authors:** Brooklyn L. Laubinger, Kelsey M. Harvey, William Isaac Jumper

**Affiliations:** 1 Prairie Research Unit, Mississippi State University, Prairie, MS 39756, USA; bls726@msstate.edu (B.L.L.); kelsey.harvey@msstate.edu (K.M.H.); 2 Department of Pathobiology and Population Medicine, College of Veterinary Medicine, Mississippi State University, Starkville, MS 39762, USA

**Keywords:** bovine, parasite control, anthelmintics, grazing management

## Abstract

Gastrointestinal nematodes (GINs) remain a significant challenge to productivity and sustainability in beef cattle systems in the United States, contributing to subclinical reductions in growth, reproductive performance, and overall herd health across production stages. Control programs have historically relied on routine anthelmintic use; however, increasing reports of anthelmintic resistance highlight the need for alternative management strategies. This narrative review synthesizes peer-reviewed literature identified through targeted searches of major scientific databases spanning approximately seven decades, with articles selected for relevance to GIN epidemiology, diagnostics, and control in beef cattle. Particular emphasis is placed on life stage-specific susceptibility, host immune development, and the role of diagnostic tools in guiding evidence-based interventions. The review further examines non-anthelmintic strategies such as grazing management, nutritional supplementation, selective breeding, and integrated parasite management practices adapted from small ruminant systems. Across studies, young and immunologically developing cattle experience the greatest productivity losses, while mature animals contribute disproportionately to pasture contamination, reinforcing the importance of targeted control measures. Overall, the literature supports a transition toward integrated, diagnostics-driven parasite control programs that sustain productivity and animal well-being while preserving long-term anthelmintic efficacy.

## Simple Summary

Gastrointestinal parasites are a common problem in beef cattle, reducing growth, milk production, and overall health. This review highlights how parasite burdens differ at various life stages, from young calves to mature cows, and how these differences affect productivity. It also examines modern diagnostic tools that can identify which parasite species are present and how heavily animals are infected. These methods allow for more targeted treatments, reducing unnecessary use of deworming drugs and helping prevent drug resistance. Understanding when and how parasites impact cattle can improve animal health, growth, and welfare. By summarizing current knowledge and practical strategies, this review provides guidance to producers and veterinarians for managing gastrointestinal parasites more effectively, supporting responsible livestock production.

## 1. Introduction

Gastrointestinal nematode (GIN) infections remain one of the most pervasive and economically important health challenges affecting beef cattle production systems worldwide [1]. By impairing nutrient utilization, altering immune function, and reducing growth, reproductive efficiency, and milk production, GIN infections contribute to substantial but often underappreciated losses in beef cattle productivity. Common GIN of pathogenic importance in cattle include members of the genera *Ostertagia*, *Cooperia*, *Trichostrongylus*, *Haemonchus*, etc. These nematodes are widely distributed and persist in diverse production environments [1].

The impact of GIN infection is not uniform across cattle populations but instead varies according to host genetics and parasite stage. Importantly, susceptibility to GIN infection varies across cattle breeds and life stages. Bos *taurus* cattle, for instance, are generally more susceptible to GIN infections than Bos *indicus* breeds, which have shown greater resistance and resilience due to their genetic adaptation to parasite heavy environments [2]. Life stage further influences GIN burden and productivity impact. Young cattle entering stocker or backgrounding operations are particularly vulnerable due to their immature, and often compromised, immune systems, resulting in measurable reductions in growth performance [3,4]. In contrast, developing heifers may experience subclinical parasitism that affects reproductive development milestones (i.e., time to puberty, time to first conception, etc.) [5]. Mature cows and bulls, while frequently asymptomatic, contribute significantly to pasture contamination, with evidence suggesting that bulls often carry disproportionately higher parasite burdens than their female counterparts [6,7].

Despite extensive research on gastrointestinal parasite control in beef cattle, much of the existing literature remains fragmented by production stage or focuses primarily on pharmacological interventions. There is a growing need for an integrated, life stage-oriented synthesis that considers how GIN burden, diagnostic approaches, and management strategies differ across the beef cattle production cycle. This need is especially relevant in regions where environmental conditions, such as warm temperatures and high humidity, promote parasite persistence and year-round transmission [8]. Additionally, increasing concerns regarding anthelmintic resistance (AR) underscore the importance of exploring non-chemical and integrated parasite control strategies tailored to specific life stages.

Hence, the objectives of this review are threefold: (1) to evaluate diagnostic practices and management strategies specific to each beef cattle life stage; (2) to synthesize current knowledge on the prevalence and impact of GIN across each stage; and (3) to identify key knowledge gaps, particularly those related to sustainable and stage-specific parasite control in beef cattle production systems.

This review synthesizes current knowledge on GIN in beef cattle using a targeted, narrative approach rather than a systematic or meta-analytic methodology. Relevant literature was identified through searches of major scientific databases, including PubMed, Web of Science, and Scopus. Searches focused on publications from approximately 1950 to the present to capture contemporary findings and foundational work. Articles were considered for inclusion if they addressed impact of GIN on production outcomes in beef cattle, diagnostic methods for GIN burden, or management and intervention strategies to control GIN burden in cattle, and contributed mechanistic, applied, or conceptual insights. Exclusion criteria were limited to non-peer-reviewed sources, publications not available in English, and studies outside the scope of this review.

## 2. Diagnostic Methods

Diagnostic testing for GIN is an important component of cattle health management in beef operations, as infection can lead to decreased productive outcomes in cattle such as decreased average daily gain (ADG), feed efficiency, and negative reproductive outcomes ultimately leading to economic loss [4,9]. Many of the economically and clinically relevant parasites of livestock are found in the gastrointestinal tract. Therefore, fecal examination is the primary method of detecting parasite infections in beef cattle [10]. Fecal parasitology tests are classified as either qualitative or quantitative. Quantitative parasitology diagnostics identify a parasite taxon by counting their diagnostic stages. These tests are the most widely used in cattle parasite diagnostics, as they convey the intensity of an infection, allowing producers to make informed management decisions. While diagnostic tests do aid in determining GIN burden, its efficacy in diagnostic sensitivity and specificity are impacted by phenomena observed in GIN behavior such as overdispersion, hypobiosis, and density dependence. Overdispersion is the concept that GIN are typically unevenly distributed among hosts, instead, a minority of animals often account for most of the parasite burden. Variation in grazing behavior and host immune competence contributes to this aggregation, which in turn affects transmission risk, diagnostic accuracy, and the effectiveness of targeted control programs [11]. With regard to hypobiosis, in several nematode species, a fraction of ingested L3 may enter arrested development at the early fourth larval stage under certain conditions. While this mechanism enhances survival during adverse environmental periods, mass emergence of arrested larvae can precipitate severe disease [12]. When cattle are exposed to high numbers of infective larvae, total worm burdens tend to increase; however, the success of individual larvae in establishing, maturing, and reproducing declines. Crowding within the gastrointestinal tract results in competition among nematodes, which limits parasite performance. These density-dependent effects are reflected by reduced establishment rates, shorter adult worm length, decreased fecundity, and higher parasite mortality. Together with overdispersion and hypobiosis, density dependence contributes to the frequently weak relationship observed between fecal egg counts (FEC) and true worm burden [13]. Therefore, understanding the type of diagnostic test utilized in determining GIN load in beef cattle is vital to making treatment decisions for the animal.

Quantitative diagnostic techniques are characterized by a flotation method. Flotation solutions allow parasitic developmental stages to float on top of a solution which has a specific gravity greater than that of the densities of the parasitic eggs. The FEC is then quantified beneath a microscope by the number of eggs that floated to the top of the solution and are reported as eggs per gram (EPG) or oocysts per gram (OPG) [14,15]. Commonly used fecal floatation solutions include sodium nitrate, saturated sucrose (commonly referred to as Sheather’s solution), and sodium chloride [16] (Table 1). The choice of fecal solution influences egg recovery and diagnostic comparability by affecting the efficiency with which parasite eggs are recovered from fecal samples [17]. Because diagnostic methods are often validated using specific flotation solutions, differences in fecal egg counts may reflect methodological variation rather than true differences in parasite burden [18]. Consequently, fecal egg count results generated using different flotation solutions are not directly comparable, underscoring the importance of methodological consistency in both research and field applications. The following sections will describe the most widely utilized diagnostic methods, outlining their respective procedures, advantages, and limitations. In contrast, qualitative diagnostic tests are used to determine the presence or absence of specific parasites. These tests can be interpreted in a semiquantitative manner by categorizing results as indicating “few,” “moderate,” or “many” parasite stages, or by using a scoring system such as 1+, 2+, or 3+, based on the reporting standards of the laboratory [10]. However, because qualitative tests are not intended to provide numerical data, such semiquantitative interpretations can be imprecise and are generally less reliable than true quantitative methods [18].

### 2.1. McMaster

The McMaster technique, while older, is the most widely utilized dilution fecal egg count flotation [19,20]. This technique is commonly used in investigations, given its low cost and relatively shorter preparation time. In this method, a fecal slurry is made by mixing a known amount of feces with a specific volume of flotation solution. Commonly, this slurry is then strained through cheese cloth or tea strainer, and the resulting liquid is used to fill a specialized counting slide, which has two gridded chambers. Strongyle eggs that are present within the gridded areas are counted and multiplied by 25, 50, or 100, depending on the protocol used [21]. A variation in the traditional McMaster technique, the high-sensitivity McMaster, employs a three-chamber slide and is more sensitive, capable of detecting as few as 8 EPG, compared to the 25, 50, or 100 EPG thresholds of the traditional method [22].

Protocols for this technique vary widely amongst laboratories and veterinary clinics, particularly in the amount of feces used, volume of flotation solution, and type of solution applied [23]. Given that this is a quantitative technique, the detection sensitivity of this diagnostic test is directly influenced by these variables. Importantly, a result showing no detectable strongyle eggs does not guarantee the absence of GIN; rather, it may indicate that egg shedding is occurring at levels below the detection limit of the test [10]. This distinction is critical when interpreting FEC results. Given that the McMaster technique has a defined detection threshold, low-intensity infections may go undetected, leading to underestimation of herd parasite burden or misinterpretation of anthelmintic efficacy [18]. Consequently, differences in diagnostic sensitivity must be considered when comparing or selecting fecal egg count methods.

### 2.2. Mini-FLOTAC

The Mini-FLOTAC technique employs a double-chambered counting disk designed to examine a total of 2 mL of fecal suspension, achieving a diagnostic sensitivity of approximately 5 EPG [24]. This dilution-based fecal egg count method involves two components: (1) the Fill-FLOTAC, a closed system that allows homogenization and filtration of the fecal suspension before directly filling the counting chamber with the filtrate, and (2) a double-chambered counting disk containing two gridded areas that are examined microscopically. The Fill-FLOTAC sampling device consists of a container, a collector, and a filter [25]. It has a conical bottom into which the fecal sample is added and weighed. Once the solution is added, the screw top has a built-in pumping device that allows for thorough homogenization. The top lid of this device also has a sieve and spout attachment that is used to fill the counting chamber [10].

This technique is user friendly and has been reported have increased accuracy, precision, and egg recovery compared to other flotation diagnostic methods [26]. When compared to the McMaster technique, Noel et al. (2017) [27] found that Mini-FLOTAC and McMaster had 83.2% and 53.7% precision, respectively. Additionally, accuracy of these tests was found to be 42.6% and 23.5% for Mini-FLOTAC and McMaster, respectively. The Mini-FLOTAC has been primarily utilized for research purposes, improving data reliability due to the method’s greater accuracy and precision in cattle [22]. Interpretation of Mini-FLOTAC results may still be influenced by sample quality, flotation solution choice, and operator experience, representing ongoing limitations of fecal egg count diagnostics.

### 2.3. Wisconsin Double-Centrifugation

The modified Wisconsin double-centrifugation method is an example of a concentration FEC method. This protocol does not involve dilution; instead, it utilizes centrifugation to achieve high detection sensitivity [28]. The Wisconsin double-centrifugation method begins with the first centrifugation step of a fecal slurry consisting of feces and water. The second centrifugation step utilizes the sediment of the first step homogenized with the flotation solution. In the absence of a dilution step, this method allows for high detection sensitivity (one EPG or less) [10].

Despite its high sensitivity, the Wisconsin method is often considered less practical. If an animal’s EPG is low enough to require this technique for detection, the parasite burden is likely not high enough to justify treatment [18]. Furthermore, compared to other quantitative methods such as the McMaster and Mini-FLOTAC, the Wisconsin technique tends to be less accurate and precise, making it a less favored option for FEC testing [22].

### 2.4. Coproculture

Genus or species-level identification of third-stage larvae (L3) based on larval morphology is achieved through the coproculture technique, which is necessary because strongyle/trichostrongyle eggs are morphologically indistinct. Coprocultures are commonly conducted using pooled fecal samples, with equal quantities of feces collected from each individual contributing to the pool. The feces are mixed with vermiculite, which serves to retain moisture and facilitate aeration, creating favorable conditions for larval development. The fecal–vermiculite mixture is incubated at room temperature or within a temperature-controlled incubator for 7 to 21 days, with a 10- to 14-day period being optimal for L3 recovery. Recovery protocols vary among laboratories. For example, Steffan et al. (1989) [29] found that inclusion of vermiculite or polystyrene in bovine coprocultures improved recovery of L3 of Ostertagia ostertagi and Cooperia oncophora. Following recovery, larvae are typically killed and stained with Lugol’s iodine to enhance the visibility of morphological features necessary for identification. Key diagnostic characteristics include total body length, structure and shape of the cephalic region, and the length and morphology of the tail and cuticular sheath [22].

Typically, 100 L3 are identified per sample; if fewer are recovered, all available larvae are examined. Results are reported as the percentage representation of each genus or species identified [30]. Coproculture is particularly useful when performed before and after a fecal egg count reduction test (FECRT), as it enables determination of the genera responsible for drug resistance [10]. However, its routine use is limited by extended incubation times and the technical expertise required for accurate larval differentiation [29].

### 2.5. Molecular Diagnostics

There is increasing interest in molecular diagnostics as a means of identification and quantification of eggs in fecal samples. These methods allow for GIN identification at the species level utilizing eggs or larvae recovered from coproculture [10]. Avramenko et al. (2015) [31] developed and validated a nemabiome sequencing approach which is based on deep amplicon next generation sequencing of the internal transcribed spacer 2 (ITS-2) rDNA locus and is equivalent to 16S rDNA sequencing of bacterial communities used in microbiome studies. This recently developed molecular diagnostic method provides accurate identification and relative quantification of GIN species within livestock host communities [31]. Within beef cattle, nemabiome metabarcoding has proven its ability to provide detailed insight into GIN parasite community structure across large sample sets, highlighting its emerging role in research, diagnostic, and surveillance efforts [32]. A major advantage of this methodology is its capacity to analyze parasite DNA from very small amounts of template that would be inadequate for conventional approaches. In diagnostic parasitology, this technology is valuable for its ability to accurately identify parasites, detect infections, and assess genetic variation [33].

### 2.6. Post-Mortem Examination

Post-mortem examination provides an opportunity to identify parasite species that may not be detected using conventional diagnostic methods. Pathological evaluation of deceased animals can reveal whether parasitic gastroenteritis represents a broader herd-level issue within an operation. Additionally, this approach assists in determining the cause of death and assessing the extent to which verminosis contributed [34]. This technique entails opening and washing specific sections of the gastrointestinal tract, followed by examination of subsamples to estimate infection intensity [33]. Although post-mortem examination can be used to diagnose GIN burden in cattle, its high cost limits its practicality for routine diagnostic or management decision-making. Nonetheless, necropsy remains a valuable research tool for characterizing the epidemiology of infections and identifying the parasite species present.

Understanding the strengths and limitations of available diagnostic methods is essential for selecting tools that align with specific management objectives. In research settings, diagnostics often prioritize accuracy and precision, favoring methods such as Mini-FLOTAC, whereas in commercial operations, cost-effective and time-efficient techniques such as the McMaster method may be more appropriate [22]. Diagnostic choice may also differ depending on whether decisions are made at the individual animal or herd level. Individual animal level decisions may justify the use of more precise methods to guide targeted interventions, while herd-level monitoring typically requires techniques that balance acceptable accuracy with affordability and throughput for large numbers of samples. Framing diagnostic selection within these decision contexts supports more practical and effective parasite management strategies.

## 3. Impact of GIN Burden Across Cattle Productive Stages

**Figure 1:**
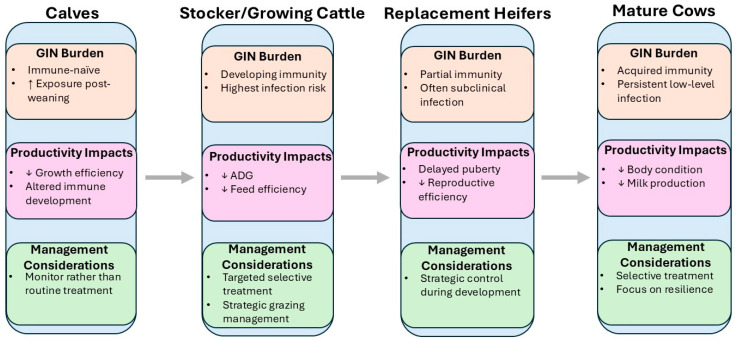
Schematic overview of GIN burden across cattle life stages, highlighting stage-specific biological status, productivity impacts, and management considerations.

Understanding how GIN burden and productive impacts vary by life stage is essential for developing targeted, stage-appropriate control strategies in beef cattle. Susceptibility to infection, immune competence, and productivity losses differ markedly between calves, growing stockers, developing heifers, mature cows, and bulls [35]. As summarized in Figure 1, the impact of GIN infection shifts across cattle life stages, with growth losses predominating in growing animals and reproductive and lactational effects becoming more relevant in mature females. These differences are driven by a combination of physiological maturity, exposure risk, and management practices, and they have direct implications for pasture contamination, treatment efficacy, and long-term herd health [13]. While diagnostic tools provide insight into infection status, interpreting GIN risk and consequences requires a life stage-specific lens [36]. This section reviews the current knowledge of GIN epidemiology, health and productivity impacts, and treatment outcomes at each stage of the beef production cycle, highlighting key vulnerabilities and management opportunities.

### 3.1. Nursing Calves

Nursing calves can be susceptible to GIN infection due to factors such as the animal’s underdeveloped immune system and grazing with an infected dam on a permanent pasture [37]. However, most infections in animals pre-weaning and before the first grazing season are subclinical, having a greater negative impact on productivity rather than overall health. Growth performance in the nursing calf is measured by weaning weight [38]. DeRouen et al. (2009) [3] conducted a study investigating the impact of treating calves for GIN on pre-weaning growth. Over the course of two years, the northern and southern beef cattle experiment stations of Louisiana grazed cow–calf pairs on previously infected pastures and compared four treatments: (1) control, no treatment (CON); (2) treatment for horn flies administered to cows only (HF); (3) anthelmintic treatment for GIN administered to calves only; and (4) treatment for horn flies administered to cows and GIN administered to calves (HF-GN). It was found that the GN treatment group presented 5–31 kg advantages in weaning weights compared with CON calves. Additionally, HF-GN treated calves responded with statistically significantly greater calf weight gains compared with CON counterparts. This study also reported that differences in ADG were significant in calves treated at 4–5 months of age, rather than 2–3 months. It is important to note that spring-born calves typically reach a greater parasite burden at 5 months of age, potentially due to increased grazing during the animal’s transition to a pasture diet [39]. Furthermore, Forbes et al. (2002) [40] reported that for beef calves under extensive grazing conditions, subclinical gastrointestinal parasitism negatively impacted the growth performance of beef calves. These authors also reported calf growth was improved in those receiving strategic anthelmintic treatment with a sustained-release ivermectin bolus compared to control cohorts. More specifically, calves receiving the sustained-release ivermectin bolus had greater ADG compared to the control calves.

The decision to implement such treatment should be guided by farm-specific considerations, including the cost-to-benefit ratio of the intervention. The economic return from increased liveweight gain must offset the cost of administering the anthelmintic [9,40]. Forbes et al. (2002) [40] investigated sub-clinical parasitism in spring born calves from the middle of their first grazing season until weaning over 3 years across four different operations. Calves either received an ivermectin sustained-release bolus in the middle of the grazing season or remained untreated controls, with FEC collected at the beginning and end of the grazing period. Although grazing management was not a primary focus of this study and statistical comparisons in treatment outcomes for different grazing systems were not reported, management practices varied among operations, including differences in stocking density and grazing strategy. Notably, calves housed in a high stocking density environment exhibited greater growth responses than those managed in at lower stocking densities. While these findings were not central to the original study objectives, they suggest that stocking density may influence GIN exposure or host response and warrant further consideration. Additionally, one operation utilizing a rotational grazing system also observed improved calf growth, further highlighting the potential interaction between grazing management and parasite dynamics. Targeted research is needed to disentangle the effects of stocking density and grazing system on GIN burden and immune development in nursing beef calves.

### 3.2. Calves Entering Stocker Operations

Weaned calves entering their first grazing season present the highest risk for GIN infection. Parasite infection in weaned calves can lead to clinical disease as well as a reduction in productivity [1,41,42,43]. A meta-analysis of the impact of Strongyle parasitism on the growth rates of young cattle (<2 years) was completed by Shephard et al. (2022) [4], and described a negative correlation between cattle ADG and FEC. Additionally, FEC differences of 100 EPG in young grazing cattle were associated with a material depression in ADG [4]. This association suggests that increases in parasite burden may adversely influence feed efficiency, a primary determinant of productive performance in stocker operations. This review did not identify a particular treatment method for parasite control, but encourages producers to come to personal management decisions depending on the cost of treatment or other interventions such as supplementing feed, compared with the costs associated with depressed growth rates and pasture larval contamination [4].

Griffin et al. (2018) [44] investigated the effect of on-arrival vaccination and deworming on stocker cattle health and growth performance. Upon arrival, stocker calves were assigned to and received one of four treatment combinations: (1) vaccinated and dewormed, (2) vaccinated and not dewormed, (3) not vaccinated and dewormed, and (4) not vaccinated and not dewormed for the 85 day trial. Calves treated with an anthelmintic at arrival exhibited lower FEC than untreated controls at day 28 (*p* < 0.0001). However, FEC also declined in non-dewormed calves over the first 56 days, and no differences in FEC were detected between dewormed and non-dewormed calves at day 56 (*p* = 0.33) or day 85 (*p* = 0.99). Vaccine treatment (*p* = 0.84), castration status (*p* = 0.88), and body weight at day 0 (*p* = 0.85) were not associated with FEC between days 28 and 85. Interestingly, deworming calves at arrival was not associated with any measured beneficial or adverse effect on health or growth performance. While this study may have lacked the power necessary to detect minute differences in health and ADG, the implication that GIN burden alone does not directly adversely impact stocker calves productivity or health is beneficial for making management decisions regarding arrivals [44].

Given that stocker calves possess limited immunological experience with GIN, it is important to consider how parasite exposure shapes the maturation of adaptive immunity in this age group. The burden of GIN impacts the bovine immune system, with acquired immunity developing slowly and often requiring prolonged exposure [35]. In cattle, protective immune responses against key parasites such as *Ostertagia ostertagi* are generally weak and take a large portion of the animal’s productive life to develop, with most calves showing measurable immunity only after extended exposure into their second grazing season [45]. Early immune mechanisms that reduce egg output or limit larval establishment may begin after several months of primary exposure, but immune responses that meaningfully restrict new infections do not typically exhibit until the second year of life [46]. The gradual and limited development of immunity indicates that these parasites may actively suppress host immune function during infection [47]. In the context of stocker calves, the delayed acquisition of adaptive immunity means that animals entering their first grazing season remain highly susceptible to reinfection and will continue to accumulate worm burdens until immunity partially develops over time. Therefore, these immunological characteristics aid in explaining the why GIN control strategies in young cattle must emphasize both burden reduction and supportive measures to protect animals during this vulnerable period.

In cattle, nutritional status of the host is an important factor in influencing the host–parasite relationship and the pathogenesis of parasite infections [48]. For example, adequate protein supplementation in the form of bypass protein or higher dietary protein improves the resilience and expression of immunity to GIN [49]. An efficacious strategy for deworming protocol is one that pairs an effective anthelmintic treatment with protein supplementation in a growing calf’s diet [50]. This strategy specifically relates to stocker calf operations, as the primary goal of this sector is to supplement for increased ADG and preparation for the feedlot [51]. Therefore, the effects of GIN burden on animal health may be easily managed by providing adequate protein supplementation, a strategy often already employed by stocker operations. It is important to note that most studies regarding how the nutritional status of the host influences parasitic infections have been done in small ruminant animals. More research is needed to further explore how increased dietary energy impacts resilience and expression of immunity to GIN.

### 3.3. Heifers

Productivity in heifer development is defined by an animal’s ability to attain puberty, conceive, and calve within the first two years of age [52]. Many factors of reproductive development are governed by management strategies, as traits associated with fertility are not highly heritable [53]. Treatment of GIN is one example of a management strategy that has a positive impact on reproduction [54]. Backes et al. (2021) [5] compared pour-on moxidectin and oxfendazole (MO), and extended release eprinomectin (ERE), and a control group on puberty attainment, reproductive parameters, and in weanling heifers over a 274-day grazing period. On d 0 and d 154, moxidectin was applied topically at 0.5 mg/kg of body weight (BW), oxfendazole was administered orally at 4.5 mg/kg of BW, and ERE was administered subcutaneously at 1.0 mg/kg of BW. Heifer BW, ADG, and body condition score (BCS) were greater in ERE groups compared with MO and untreated heifers. Additionally, overall pregnancy rates were greater in ERE compared with heifers that did not receive treatment throughout the trial [5]. This indicates that ERE is an effective treatment strategy for developing replacement heifers with regard to BW, ADG, BCS, and pregnancy rates compared to no treatment or MO. Increased ADG leads to hastened puberty, allowing more cycles before breeding. It is ideal for a heifer to cycle 1–2 times before breeding, as it significantly improves both conception rates and pregnancy retention [55]. Therefore, the negative impact that GIN infection has on ADG may also inhibit reproductive development, and heifer productivity.

Contrarily, Loyacano et al. (2002) [56] did not find similar results when comparing pregnancy rates between heifers treated for GIN and untreated heifers. Over a four year trial conducted at the Dean Lee Research Station in Alexandria, LA, authors investigated the effect of GIN (primarily Ostertagia ostertagi) and liver fluke (Fasciola hepatica) infections on weight gain and reproductive performance of weaned beef heifers. This trial compared an untreated control group, treatment for GIN only, treatment for liver flukes only, and treatment for both Gin and liver flukes. A total of seven treatments were administered throughout the developmental period post-weaning until pregnancy palpation. This study found that heifers that did not receive GIN treatment had lower BW and BCS at breeding compared to the two groups that did receive treatment for GIN. However, heifers treated for GIN alone did not increase pregnancy rates compared to the untreated control group [56]. Further research is warranted to better understand the dynamics between GIN burden post-weaning and reproductive development in beef heifers.

### 3.4. Cows

The mature cow herd is a major contributor to pasture contamination [57]. This contamination intensifies throughout the grazing season due to auto-infection cycles, where cows ingest infective L3 from herbage and subsequently shed eggs from maturing adult worms within the gastrointestinal tract [13]. In cows, GIN infections are associated with decreased ADG, diminished reproductive efficiency, and reductions in milk yield [6,58].

Recognizing the importance of parasite control in managing cow herd productivity, producers must evaluate the most effective and practical deworming strategies for their operations. Johnson et al. (2020) [6] demonstrated that ERE reduced FEC and improved both BCS and reproductive performance in beef cows compared to short-acting pour-on ivermectin. Although the initial cost of long-acting treatments may be higher, these benefits can translate into economic gains in herds where infrequent handling makes repeated deworming impractical.

Furthermore, there is evidence to suggest that there is an association between GIN burden, stress hormones, and reproductive outcomes. Kasimanickam et al. (2021) [59] reported that cows treated with anthelmintics exhibited elevated progesterone concentrations and a shift in immune response toward anti-inflammatory Th2 cytokines, which support pregnancy maintenance [60]. In contrast, untreated pregnant cows showed higher levels of stress hormones, lower conception rates, and increased expression of pro-inflammatory Th1 cytokines, which have been implicated in impaired embryo development and early pregnancy loss [59,61]. Elevated blood cortisol may initially serve a protective role by modulating immune responses to limit tissue damage caused by parasites. However, sustained elevation of circulating cortisol can be detrimental, compromising host immunity, enhancing susceptibility to parasitic infection, and facilitating parasite survival and reproduction [62]. Stromberg at al. (1997) [63] found similar reproductive performance results when comparing strategic anthelmintic treatment with an untreated control group. Across two years, the treated cow–calf group was given fenbendazole at turnout and at midsummer, while the control group remained untreated. This study found that treated cows exhibited a significant increase in reproductive performance, with the pregnancy rate being 94% for treated cows compared to 82% for the control group [64].

Taken together, these studies demonstrate that mature cows play a central role in maintaining gastrointestinal nematode transmission within grazing systems and that even subclinical infections can negatively affect reproductive performance and physiological status. Strategic anthelmintic treatment has been shown to reduce fecal egg shedding, improve body condition and endocrine profiles supportive of pregnancy, and increase pregnancy rates in beef cows. These findings underscore the importance of effective parasite control in optimizing cow herd productivity under grazing conditions.

### 3.5. Bulls

As mature bulls are not typically housed with the cow herd year-round, they can easily be overlooked when developing a parasite control program. However, sex-based differences in susceptibility to GIN have been observed across multiple ruminant species, including cattle [63]. In a foundational study by Herd et al. (1992) [65], bulls exhibited higher FEC compared to steers and heifers when co-grazing on the same contaminated pasture, suggesting a greater parasite burden or increased worm fecundity in intact males. While steers and heifers did not differ in egg counts, the consistent elevation in bulls implies a sex-related physiological basis for the observed differences in parasitism. The identification of sex-related differences in worm susceptibility suggests that estrogens may enhance heifers’ immune responses to nematodes, whereas testosterone may suppress immune function in males [66]. These findings highlight the importance of including sex as a biological variable in both research design and herd-level parasite management strategies.

Despite these insights, significant knowledge gaps remain with regard to the sex-related susceptibility of bulls to GIN [13]. Increased susceptibility in bulls may contribute to higher levels of pasture contamination and reduced health or productivity in untreated individuals [65]. Further research is needed to clarify the contribution of bulls to pasture contamination relative to other sex classes, as the existing literature is limited and outdated. Additionally, future studies should evaluate whether gastrointestinal nematode infections measurably affect key productivity indicators in bulls, including fertility and weight gain, as well as their potential role in surveillance programs and the effectiveness of pre-breeding treatment strategies.

## 4. Prevention and Treatment Methods

### 4.1. Anthelmintics

Anthelmintic drugs are commonly utilized in beef cattle production in the treatment and prevention of internal parasite infections [67]. Several classes of anthelmintics exist that are differentiated based on chemical structure and mode of action. These include both broad-spectrum and narrow-spectrum groups. Broad-spectrum anthelmintics commonly used in ruminants can be categorized into three groups: (1) benzimidazoles, (2) imidazothiazoles and tetrahydropyrimidines, and (3) avermectins and milbemycins [68]. These broad-spectrum anthelmintics are effective against majority of the roundworm species reported in cattle. Narrow-spectrum anthelmintics provide targeted activity against specific parasite groups and are primarily represented by the salicylanilide and substituted phenol classes [67]. These products have historically offered reliable reductions in parasite burden, improvements in weight gain, and enhanced reproductive performance [3,6,40]. As a result, many operations have adopted routine, whole-herd deworming programs, often administered at fixed intervals rather than based on diagnostics or grazing conditions. While effective in the short term, these blanket treatments apply strong selection pressure on parasite populations, accelerating the development of drug-resistant GIN species [69].

Anthelmintic resistance is the heritable decline in a parasite population’s responsiveness to a drug to which it was previously susceptible. Anthelmintic resistance has accelerated as nematodes that survive treatment continue to reproduce, progressively reducing drug efficacy. Resistance has now been documented across multiple helminth species, animal hosts, and major anthelmintic drug classes on nearly every continent [69]. In beef cattle, there have been increasing numbers of reports of anthelmintic resistance in GIN [70]. Within the US at the present time, few studies have been published establishing the prevalence of resistance of parasites in cattle. However, the studies that have been published have documented that there is widespread and increasing resistance of GIN in beef cattle, leading to the call for a new direction in treatment methods whether that be new drug classes or non-chemical measures.

Combination therapy, using two drugs concurrently that are from two different classes of anthelmintic, has become a rising practice throughout the beef cattle industry [71]. Additionally, treating with two different anthelmintic classes should aid in slowing further development of resistant parasites in cattle [72]. The first commercially available combination therapy is a combination of levamisole and doramectin, under the tradename Valcor^®^ [73]. Studies conducted in Australia and New Zealand have found that this combination product is highly effective in treating cattle for economically important GINs [71,74]. This product demonstrated ≥99.7% efficacy within the Australia study and ≥99.9% efficacy in New Zealand cattle [71,74]. These results show that commercially available combination anthelmintic treatment presents an option for producers to treat GINs chemically, while also aiding in slowing down anthelmintic resistant parasite reproduction. While current results of combinations anthelmintics are promising and effective against resistant nematodes, there remains concern that overuse of these products in management settings will hasten multiple drug resistance.

### 4.2. Rotational Grazing and Pasture Management

Alternative methods to GIN control are becoming necessary due to anthelmintic resistance [75]. Additionally, there is an increasing consumer desire for reduced use of pharmaceuticals in livestock as well as decreased chemical residues in food and the environment [76]. Rotational grazing is a management strategy aimed at reducing cattle exposure to GIN larvae on pasture. This approach moves animals among forage plots, with grazing intervals determined by land availability and forage conditions and can provide several production benefits [77]. However, one study in cattle reported that rotational grazing alone forced animals to graze more closely to fecal pats, resulting in no improvement in GIN control [78]. Smith et al. (2009) [79] investigated the effects of grazing management on livestock exposure to parasites by comparing a set-stock grazing system with rotational grazing systems of varying intensity (size of pasture and number of rotations). It was found that cattle in the rotational grazing systems interacted more closely/often with fecal pats than that of the set-stock group. This interaction increased concurrently with the intensity of the rotational grazing system, as with a higher stocking density, cattle graze closer to the ground and fecal pats are not as spread out as in conventional stocking, so that exposure is increased over time [79]. However, this study focused on grazing behavior and did not collect any measure of productivity or FEC to determine if the grazing systems had a true impact on parasite burden. In contrast, when rotational grazing was integrated with complementary measures, such as strategic anthelmintic treatment, reductions in parasitism were observed [80]. Further research is needed to elucidate the effects of rotational grazing on gastrointestinal nematode burden in cattle, as most robust evidence currently originates from small ruminant systems, with comparatively limited data available for cattle.

### 4.3. Selective Breeding

For most of evolutionary history, host populations have relied on natural selection to favor individuals better able to withstand or limit GIN infections. This process still acts in wild small ruminants, helping maintain population health and stability. In domestic herds, however, the widespread adoption of anthelmintics over the past five decades has disrupted this evolutionary pressure by artificially suppressing worm populations. As drug resistance has become widespread and concerns over sustainability and animal welfare have grown, breeding livestock for improved resistance or resilience to GINs has emerged as a critical long-term strategy [75].

It is important to distinguish parasite resistance, defined as the ability to limit parasite establishment or fecundity, from resilience, which reflects the capacity to maintain performance despite infection [81]. In cattle, Bos *indicus* breeds are known to be more resilient to the effects of parasite burden than Bos *taurus* breeds. Therefore, a common strategy for selective breeding in the southern US where climate allows such management practices, is to select for Bos *indicus* influenced cattle in order to strengthen host immunity [82]. Crossbred cattle with Bos *indicus* influence have been shown to exhibit greater resilience to infection and in some cases, lower infection levels compared with Bos taurus breeds [83].

Consequently, genetic selection for resistance or resilience is increasingly viewed as a cornerstone of integrated parasite management strategies that reduce reliance on anthelmintics while maintaining animal health and productivity. While genetic resistance to GINs has been extensively researched in small ruminants, comparatively fewer long-term selection studies exist in cattle, highlighting a need for continued research to optimize breeding strategies in beef systems.

## 5. Conclusions

The burden of GIN remains a significant health and productivity challenge across all life stages of beef cattle. Diagnostic methods have advanced to provide more accurate estimations of parasite burden, allowing for more informed treatment decisions. However, despite evidence supporting the benefits of strategic anthelmintic use, the beef industry continues to face rising concerns regarding drug resistance, limited efficacy of single-treatment approaches, and sustainability of long-term chemical control.

This review highlights that most current research has centered on pharmacological interventions and their impacts on productivity, immunity, and reproductive performance. Yet, relatively little attention has been given to the potential of alternative or integrated parasite management strategies, particularly grazing management practices such as pasture rotation, rest periods, and multi-species grazing, to reduce parasite exposure and pasture contamination in beef cattle. These approaches may offer long-term, sustainable control by disrupting parasite life cycles and minimizing reinfection pressure.

Further research is needed to explore the efficacy of these non-chemical strategies, both independently and in combination with targeted anthelmintic use. Identifying grazing systems that effectively limit GIN transmission could reduce reliance on dewormers, enhance animal resilience, and support the sustainability of beef cattle production systems.

## Figures and Tables

**Table 1:** Solutions commonly utilized for flotation methods in fecal egg count diagnostics, including specific gravity and common method associated with the solution.

Solution	Specific Gravity	Methods
Sodium Nitrate	1.25–1.3	McMaster, Mini-FLOTAC
Saturated Sodium Chloride	1.2	McMaster
Zinc Sulfate	1.18	Wisconsin Double Centrifugation for *Giardia*
Saturated Sucrose (Sheather’s)	1.25	Wisconsin Double Centrifugation
Saturated Sugar	1.33	Wisconsin Double Centrifugation

## Data Availability

No new data were created or analyzed in this study. Data sharing is not applicable to this article.

## Abbreviations

The following abbreviations are used in this manuscript:

GIN	Gastrointestinal nematodes
AR	Anthelmintic resistance
ADG	Average daily gain
FEC	Fecal egg count
EPG	Eggs per gram
OPG	Oocysts per gram
CON	Control
HF	Horn flies
HF-GN	Horn flies and gastrointestinal nematodes
MO	Moxidectin and oxfendazole
ERE	Extended-release eprinomectin
BW	Body weight
BCS	Body condition score
